# Theseus: fast and optimal affine-gap sequence-to-graph alignment

**DOI:** 10.1093/bioinformatics/btag561

**Published:** 2026-07-27

**Authors:** Albert Jiménez-Blanco, Lorién López-Villellas, Juan Carlos Moure, Miquel Moreto, Santiago Marco-Sola

**Affiliations:** Department of Computer Science, Universitat Politècnica de Catalunya, Barcelona, 08034, Spain; Computer Sciences Department, Barcelona Supercomputing Center, Barcelona, 08034, Spain; Department of Computer Science and Systems Engineering, University of Zaragoza, Zaragoza, 50018, Spain; Department of Computer Architecture and Operative Systems, Universitat Autònoma de Barcelona, Barcelona, 08193, Spain; Computer Sciences Department, Barcelona Supercomputing Center, Barcelona, 08034, Spain; Department of Computer Architecture, Universitat Politècnica de Catalunya, Barcelona, 08034, Spain; Department of Computer Science, Universitat Politècnica de Catalunya, Barcelona, 08034, Spain; Computer Sciences Department, Barcelona Supercomputing Center, Barcelona, 08034, Spain

## Abstract

**Motivation:**

Sequence-to-graph alignment is a central problem in bioinformatics, with applications in multiple sequence alignment (MSA) and pangenome analysis, among others. However, current algorithms for optimal affine-gap alignment impose high memory and computational requirements, limiting their scalability to aligning long sequences to complex graphs. Practical solutions partially address this problem using heuristic strategies that ultimately trade off optimality for speed.

**Results:**

This work presents Theseus, a novel, fast, and optimal affine-gap sequence-to-graph alignment algorithm. Theseus leverages similarities between genomic sequences to accelerate the alignment computation and reduces the overall memory requirements without compromising optimality. To that end, Theseus processes only a subset of the dynamic programming cells, using a sparse-data strategy that enables efficient sequence-to-graph alignment. Moreover, our algorithm supports optimal affine-gap alignment on arbitrary directed graphs, including those with cycles. We evaluate Theseus on two key problems: MSA and pangenome read mapping. For MSA, we compare it against SPOA, abPOA, and POASTA. Theseus is 1.6× to 17.6× faster than POASTA, and 7.3× faster, on average, than SPOA, both optimal aligners. Compared with abPOA, Theseus ensures optimality and scales to the largest problems. For pangenome read mapping, we benchmark Theseus against the alignment stage of the mapping tool vg map, along with the alignment kernels of SPOA, abPOA, and POASTA. Theseus outperforms the other methods, showing a 1.9× to 16.9× speedup on short reads. Moreover, Theseus is 1.5× to 36.3× faster than vg when aligning against synthetic cyclic graphs.

**Availability and implementation:**

Theseus code and documentation are publicly available at https://github.com/albertjimenezbl/theseus-lib.

## 1 Introduction

Sequence-to-graph alignment is a central problem in modern bioinformatics (Eizenga *et al.* 2020) driven by the growing use of sequence graphs to compactly model genetic diversity (Computational Pan-Genomics Consortium 2018, Miga and Wang 2021) and reduce reference bias (Garrison *et al.* 2018). Consequently, sequence-to-graph alignment has applications across multiple fields, including comparative genomics (Tettelin *et al.* 2008), evolutionary biology (Zhang *et al.* 2019), and pangenomics (Liao *et al.* 2023), among others (Miller *et al.* 2010, Compeau *et al.* 2011, Marco-Sola *et al.* 2012, Marco-Sola and Ribeca 2015), enabling fundamental tasks such as multiple sequence alignment (MSA) (Lee *et al.* 2002) and read mapping to pangenome graphs (Rautiainen and Marschall 2020).

Multiple sequence alignment (MSA) is a fundamental analysis in bioinformatics and genomics (Gotoh 1999), providing the basis for studying conservation, variation, and evolutionary relationships across sequences. To that end, Partial Order Alignment (POA) (Lee *et al.* 2002) is a widely used method that progressively builds an alignment of a set of *N* sequences. In its original formulation, POA provides an efficient and informative solution to the MSA problem (Lee *et al.* 2002), progressively building a partial-order graph that represents the incremental multiple alignment. This is done by iteratively aligning each new sequence $S_{i}$ to the current sequence-graph and then updating the graph to incorporate each newly computed alignment.

Similarly, pangenome read mapping has emerged as a fundamental problem in bioinformatics, crucial for analyzing genetic variation across populations by replacing traditional linear references with pangenome graphs. Initiatives such as the Human Pangenome Reference Consortium (Liao *et al.* 2023) and the 1000 Genomes Project (1000 Genomes Project Consortium *et al.* 2015) reflect the growing interest in efficiently modeling genetic diversity and reducing reference bias in the analysis of highly variable regions, such as the Major Histocompatibility Complex (MHC) (Chin *et al.* 2020). Similar to traditional read-mapping tools, most currently used pangenome read mappers (Garrison *et al.* 2018, Rautiainen and Marschall 2020) implement the seed-and-extend paradigm. This paradigm consists of two phases: (i) A seeding strategy is used to find candidate mapping locations, and (ii) these candidates are extended using an alignment algorithm to determine the better-scoring locations in the reference pangenome graph. Current pangenome read mappers usually work with directed acyclic graphs (DAG) (Garrison *et al.* 2018) or admit small cycles (Rautiainen and Marschall 2020), but do not directly support large cycles. VG implicitly represents such cycles by unfolding the cyclic components in the reference pangenome. However, this unfolding process can substantially increase the size of the unfolded graph with respect to its cyclic counterpart (Kavya *et al.* 2019).

However, the large scale of sequence-graphs used for MSA and pangenome analyses, combined with the ever-growing volume of genomic data (Goodwin *et al.* 2016), places significant stress on traditional sequence-to-graph alignment algorithms (Computational Pan-Genomics Consortium 2018, López-Villellas *et al.* 2023, 2024). Even for the simplified version of DAGs, the algorithmic cost of traditional algorithms grows proportionally to the product of the number of bases in the reference graph and the length of the aligned sequence (Jain *et al.* 2020). The problem becomes harder when considering cyclic graphs. As a result, traditional methods require high memory and runtime and scale poorly when dealing with large numbers of sequences or complex, large pangenome graphs (Darby *et al.* 2020).

Classical solutions rely on extending the dynamic programming (DP) formulation of sequence-to-sequence alignment (Needleman and Wunsch 1970) to sequence-graphs. Navarro introduced the first such extension, proposing a DP algorithm that computes the optimal alignment of a sequence to an arbitrary graph for approximate hypertext matching (Navarro 2000). This foundational algorithm was later extended to support arbitrary costs (Jain *et al.* 2020). However, its time and memory demands make it impractical for large-scale genomic data, which has motivated the proposal of multiple constant-factor optimizations, such as SIMD vectorization and bit-parallel techniques (Myers 1999, Doblas *et al.* 2023, 2025b). Notably, SPOA (Vaser *et al.* 2017) is a widely used state-of-the-art POA library for computing MSA that speeds up the traditional DP computation through SIMD vectorization. Similarly, GraphAligner (Rautiainen *et al.* 2019, Rautiainen and Marschall 2020) is a widely used pangenome read-mapping tool that speeds up edit-distance computation through bit-parallel techniques.

In contrast to optimal approaches, many practical implementations employ aggressive heuristics to accelerate computation and reduce memory usage at the expense of accuracy, often missing the optimal alignment solution. Examples of such heuristic strategies include the banded alignment mode of GraphAligner (Rautiainen and Marschall 2020), the seed-chaining approach of minigraph (Li *et al.* 2020), and the adaptive banding in abPOA (Gao *et al.* 2021).

More recently, new output-sensitive methods (Doblas *et al.* 2025a) have been proposed whose runtime scales with the difficulty of the alignment (i.e. alignment score), adjusting to the workload complexity. Notably, the Wavefront Alignment algorithm (WFA) (Marco-Sola *et al.* 2021, 2023, Aguado-Puig *et al.* 2023, Haghi *et al.* 2023, Alonso-Marín *et al.* 2024) introduced an output-sensitive formulation that achieves optimal sequence-to-sequence alignment, but it is limited to linear references. Other methods, inspired by classical informed searches (Hart *et al.* 1968), implement $A^{*}$-based strategies to reduce the number of DP cells explored in graph alignment. POASTA (van Dijk *et al.* 2024) and Astarix (Ivanov *et al.* 2020) follow this strategy, reducing DP cell computations at the expense of overheads for maintaining a priority queue, computing the heuristic function, and requiring additional memory.

In this work, we present Theseus, a fast and memory-efficient output-sensitive algorithm for optimal affine-gap sequence-to-graph alignment. Inspired by the WFA algorithm for sequence-to-sequence alignment (Marco-Sola *et al.* 2021, 2023), Theseus exploits the similarity between sequences to accelerate computations and reduce the memory footprint. Unlike other sequence-to-graph alignment algorithms tailored for specific use cases, Theseus is flexible enough to be applied to many sequence-to-graph alignment applications. Moreover, Theseus works on directed graphs and supports cycles, efficiently addressing the sparse nature of graph exploration.

We evaluate Theseus on two fundamental and critical applications: MSA computation and pangenome read mapping. First, for MSA computation, we compare Theseus against the state-of-the-art POA methods SPOA and POASTA (exact) and abPOA (heuristic). Our results show that Theseus is 1.6× to 17.6× faster than POASTA, and 7.3× faster, on average, than SPOA. In the case of abPOA, its speed comes at the cost of optimality, whereas Theseus always produces optimal alignments and, unlike abPOA, scales to the largest problems instead of exhausting memory. Second, for read mapping, we compare Theseus against the sequence-to-graph alignment kernels in VG map, SPOA, abPOA, and POASTA. In this experiment, Theseus outperforms the other methods, showing a 1.9× to 16.9× speed improvement on short reads. Moreover, we compare Theseus against vg when aligning sequences to synthetic cyclic graphs. We observe that Theseus is 1.5× to 36.3× faster.

In summary, this work makes the following contributions.

- We present Theseus, a novel output-sensitive algorithm for fast and memory-efficient computation of optimal affine-gap sequence-to-graph alignment that supports reference graphs with cycles.
- We propose a diagonal invalidation strategy that compactly represents and updates the set of invalid alignment diagonals, eliminating redundant computations during sequence-to-graph alignment.
- We introduce sparse wavefronts, an efficient data structure to store intermediate wavefronts over sequence-graphs. We also propose a sequence-graph unified coordinate system to efficiently operate over multiple sparse wavefronts across the vertices of a sequence-graph.
- We introduce a memory-efficient backtrace algorithm to recover the optimal alignment path from sparse wavefronts over sequence-graphs.
- We provide a high-performance, open-source implementation of Theseus, distributed as both a library and two standalone minimal tools, publicly available to the community. The first tool, *theseus_msa* allows to compute the MSA of a set of input sequences. The second, *theseus_aligner* allows user to align sequences to a reference graph, given the starting alignment positions.

## 2 Background

A sequence-graph is a tuple G=(V,E,σ), where *V* denotes a set of vertices, E⊆V×V its set of directed edges, and $\sigma :V\to \Sigma^{*}$ assigns a sequence of symbols over alphabet Σ to every vertex. For simplicity, we denote the length of the sequence associated with *v* as |*v*|. Given vertices s,t∈V, a path *P* from *s* to *t* is defined as the ordered sequence of vertices $\left(s,v_{1},\ldots ,v_{k},t\right)$ connected by edges in the sequence-graph *G*. Any path $P=(s,v_{1},\ldots ,v_{k},t)$ has an associated sequence-path $S_{P}=\sigma (s)\cdot \sigma (v_{1})\ldots \sigma (v_{k})\cdot \sigma (t)$ obtained by concatenating the sequences of its vertices. Let *Q* be a sequence of length *m*, which we name *query*, and let *G* be a sequence-graph, which we name *graph-reference*. Then, the optimal sequence-to-graph alignment is the sequence of four basic operations (i.e. match, mismatch, insertion, and deletion) that transform *Q* into a sequence $S_{P}$, spelled by a path in *G* originating at some vertex *s*, while minimizing a given score function.

Most practical applications employ score function models: gap-linear and gap-affine. The first is the simple gap-linear score function (also known as weighted edit distance), which assigns fixed penalty values (a,x,e) for matches, mismatches, and gap extensions (applied to both insertions and deletions), respectively. The second model is the affine-gap score function (Gotoh 1982), defined as a tuple (a,x,o,e) of four values, where *a* is the cost of a match, *x* is the cost of a mismatch, *o* is the cost of opening (starting) a gap, and *e* is the cost of extending it. This formulation models the fact that starting a new gap is more expensive than extending an existing one. For example, two insertions interrupted by another operation require paying two gap-opening and two extension penalties 2o+2e, while a single uninterrupted two-base gap costs only o+2e. Using a gap-affine function allows treating long gaps as a single event rather than multiple independent edits, yielding more accurate biological interpretations.

The optimal sequence-to-graph alignment can be computed using the recurrence equations in Equation (1). $I_{v,i,j}$ and $D_{v,i,j}$ represent the optimal score to align the prefix Q[1, *i*] against any valid path that reaches offset *j* of vertex *v* and ends in an insertion or a deletion, respectively. The third value, $M_{v,i,j}$, represents the optimal alignment score of reaching position (v,i,j), regardless of the last edit operation. The initial conditions (j=0) at each vertex *v* depend on the last scores computed at every vertex *u* (j=|u|) with an incoming edge to *v*; i.e. (u,v)∈E. Here, $s(Q_{i},\sigma {\left(v\right)}_{j})=a$ if $Q_{i}=\sigma {\left(v\right)}_{j}$ (match) and $s(Q_{i},\sigma {\left(v\right)}_{j})=x$ otherwise (mismatch).


(1) $$\begin{array}{c} I_{v,i,j}=\{\begin{array}{cc} \min\{I_{u,i,|u|}\},\forall (u,v)\in E & \;if\;j=0\\ \min\{M_{v,i,j-1}+o+e,I_{v,i,j-1}+e\} & \;if\;j>0 \end{array}\\ D_{v,i,j}=\{\begin{array}{cc} \min\{M_{v,i-1,j}+o+e,D_{v,i-1,j}+e\} & if\;j\geq 0 \end{array}\\ M_{v,i,j}=\{\begin{array}{cc} \min\{M_{u,i,|u|}\},\forall (u,v)\in E & \;\;\;\;\;\;\;\;\;\;if\;j=0\\ \min\left\{\begin{array}{c} I_{v,i,j}\\ D_{v,i,j}\\ M_{v,i-1,j-1}+s(Q_{i},\sigma {\left(v\right)}_{j}) \end{array}\right\} & \;\;\;if\;j>0 \end{array} \end{array}$$

In practice, solutions to Equation (1) are computed using DP, as the problem exhibits optimal substructure where every partial solution of an optimal alignment is optimal. Common implementations under the affine-gap model allocate three DP matrices per vertex *v* ($M_{v}$, $I_{v}$, and $D_{v}$) of dimension O(m|v|). This results in O(m(L+|E|)) time and space complexity (Navarro 2000), with $L=\sum_{v\in V}|v|$ the total length of the sequences in the graph.

### 2.1 Wavefront alignment algorithm

For the sequence-to-sequence alignment problem, the WFA Algorithm is an output-sensitive algorithm that leverages sequence similarity to accelerate the alignment computation. Unlike traditional DP-based algorithms, which require computing the full DP matrix, WFA evaluates DP cells in order of increasing alignment score until the optimal solution is found. Specifically, WFA computes partial alignments for increasing values of score, from s=0, until the optimal alignment score $s^{*}$.

The WFA algorithm relies on two key concepts: diagonal encoding and furthest-reaching cells. First, to compute the optimal alignment between two sequences *Q* and *T*, any DP cell (i,j) in the matrix can be uniquely identified by its *diagonal* k=j−i and its offset off=i, which specifies the position of the cell along that diagonal. This defines a coordinate transformation from (i,j) to (k,off), where the original indices can be recovered as (i,j)=(off,k+off). Second, for a given score *s* and diagonal *k*, the *furthest-reaching cell* $F_{s,k}$ represents the cell with score *s* on diagonal *k* with the most advanced offset *off* along the diagonal. The vector of all furthest-reaching cells across all diagonals for a given score *s* is called the *wavefront* at score *s*. Focusing only on the furthest-reaching cells for each score and diagonal, WFA avoids computing the full DP matrix, resulting in significant efficiency gains for similar sequences.

Under the affine-gap score model, WFA computes the optimal sequence-to-sequence alignment using the recurrence relations in Equation (2). To that end, WFA maintains three wavefront data structures, $\tilde{M}$, $\tilde{I}$, and $\tilde{D}$, which record all the wavefronts for each score *s*, corresponding to the match-mismatch state, the insertion state, and the deletion state of the DP formulation, respectively. The algorithm proceeds iteratively by score, alternating between two phases: *next* and *extend*. In the *next* phase, for each score *s*, the algorithm computes the initial offsets of ${\tilde{M}}_{s}$, ${\tilde{I}}_{s}$, and ${\tilde{D}}_{s}$ for all reachable diagonals by applying Equation (2) using previously computed wavefronts. Then, during the *extend* phase, each initial furthest-reaching cell in the ${\tilde{M}}_{s}$ wavefront is advanced following consecutive matches along the diagonal (i.e. computing the Longest Common Prefix, LCP, between the corresponding suffixes of the query *Q* and the text *T*). The algorithm terminates when it first meets the alignment’s end condition, immediately identifying the optimal score $s^{*}$.


(2) $$\begin{array}{c} {\tilde{I}}_{s,k}=\max\left\{\begin{array}{cc} {\tilde{M}}_{s-o-e,k-1} & \left(\text{Open}\;\text{insertion}\right)\\ {\tilde{I}}_{s-e,k-1} & \left(\text{Extend}\;\text{insertion}\right) \end{array}\right\}\\ {\tilde{D}}_{s,k}=\max\left\{\begin{array}{cc} {\tilde{M}}_{s-o-e,k+1} & \left(\text{Open}\;\text{deletion}\right)\\ {\tilde{D}}_{s-e,k+1} & \left(\text{Extend}\;\text{deletion}\right) \end{array}\right\}+1\\ {\tilde{M}}_{s,k}=\max\left\{\begin{array}{cc} {\tilde{M}}_{s-x,k}+1 & \left(\text{Substitution}\right)\\ {\tilde{I}}_{s,k} & \left(\text{Insertion}\right)\\ {\tilde{D}}_{s,k} & \left(\text{Deletion}\right) \end{array}\right\}+\mathrm{LCP}(Q,T) \end{array}$$

As a result, WFA computes *s* wavefronts of increasing size and evaluates an LCP() operation in each of them. Each diagonal offset can only advance up to the length of the sequences. Thus, the time complexity of WFA is O(ns) when the sequences are similar (s≪n).

## 3 Methods

In this section, we describe our method, Theseus, to compute fast and optimal affine-gap sequence-to-graph alignment. We begin by generalizing the notion of furthest-reaching cells to sequence-graphs and deriving the corresponding recurrence relations (Section 3.1). Then, we present the Theseus algorithm, highlighting the particular challenges that arise when aligning against a sequence-graph reference. (Section 3.2). Next, we introduce a diagonal invalidation strategy that avoids redundant work by tracking explicitly and implicitly explored diagonals (Section 3.3). Then, we describe a unified coordinate system that enables the efficient combination and processing of sparse wavefronts from different vertices (Section 3.4). Finally, we present a memory-efficient backtrace method to reconstruct the optimal alignment path from the sparse wavefront representation (Section 3.5).

### 3.1 Furthest-reaching cells in sequence-graphs


(3) $$\begin{array}{c} {\tilde{I}}_{v,s,k}=\max\left\{\begin{array}{c} {\tilde{M}}_{v,s-o-e,k-1}\\ {\tilde{I}}_{v,s-e,k-1}\\ {\tilde{I}}_{u,s,k}\;(\text{Jump}) \end{array}\right\}\\ \text{where}\;(u,v)\in E\wedge {\tilde{I}}_{u,s,k}=|u|-k\\ {\tilde{D}}_{v,s,k}=\max\left\{\begin{array}{c} {\tilde{M}}_{v,s-o-e,k+1}\\ {\tilde{D}}_{v,s-e,k+1} \end{array}\right\}+1\\ {\tilde{M}}_{v,s,k}=\max\left\{\begin{array}{c} {\tilde{M}}_{v,s-x,k}+1\\ {\tilde{I}}_{v,s,k}\\ {\tilde{D}}_{v,s,k}\\ {\tilde{M}}_{u,s,k}\;(\text{Jump}) \end{array}\right\}+\mathrm{LCP}(Q,\sigma (v))\\ \text{where}(u,v)\in E\wedge {\tilde{M}}_{u,s,k}=|u|-k \end{array}$$

Theseus generalizes the core concepts of the WFA algorithm to the broader problem of sequence-to-graph alignment, progressively exploring the solution space by computing partial alignments with increasing score. For that, Theseus computes successive wavefronts of furthest-reaching cells using Equation (3).

We propose to work with three vertex-local wavefront structures ${\tilde{M}}_{v}$, ${\tilde{I}}_{v}$, and ${\tilde{D}}_{v}$ for each vertex v∈V, instead of using three global structures for all vertices. In this way, for each score *s*, vertex *v*, and diagonal *k*, Theseus computes the furthest-reaching cell as the maximum over the possible previous states reaching the position (v,s,k) in ${\tilde{M}}_{v,s,k}$, ${\tilde{I}}_{v,s,k}$, and ${\tilde{D}}_{v,s,k}$.

However, note that the recurrences on Equation (3) are not vertex-local, as their values may depend on wavefront values from adjacent vertices in the sequence graph. This requires redefining the recurrence equations to account for jumps across vertices, ensuring that the furthest-reaching cells at score *s* include contributions from incoming wavefronts. Note that a diagonal *k* in vertex *v* can be reached in two different ways: either from a diagonal in the same vertex *v* or from a diagonal of an incoming vertex *u* s.t. (u,v)∈E. Therefore, ${\tilde{I}}_{v,s,k}$ and ${\tilde{M}}_{v,s,k}$ must also account for the offsets propagated from each incoming vertex *u* where the diagonal *k* in *u* has an offset value of |u|−k; that is, ${\tilde{I}}_{u,s,k}=|u|-k$ and ${\tilde{M}}_{u,s,k}=|u|-k$, respectively.

### 3.2 Theseus algorithm

Theseus’ algorithm explores the solution space in increasing order of score *s* using Equation (3). It starts at an initial vertex $v_{0}$ with score s=0 and continues until it reaches a path in *G* that yields a complete alignment of *Q*. Since this is the first complete alignment of *Q* found during its score-ascending search, it is necessarily optimal and achieves the minimal alignment score $s^{*}$. Algorithm 1 presents the overall structure of the Theseus algorithm.

**Figure 1:**
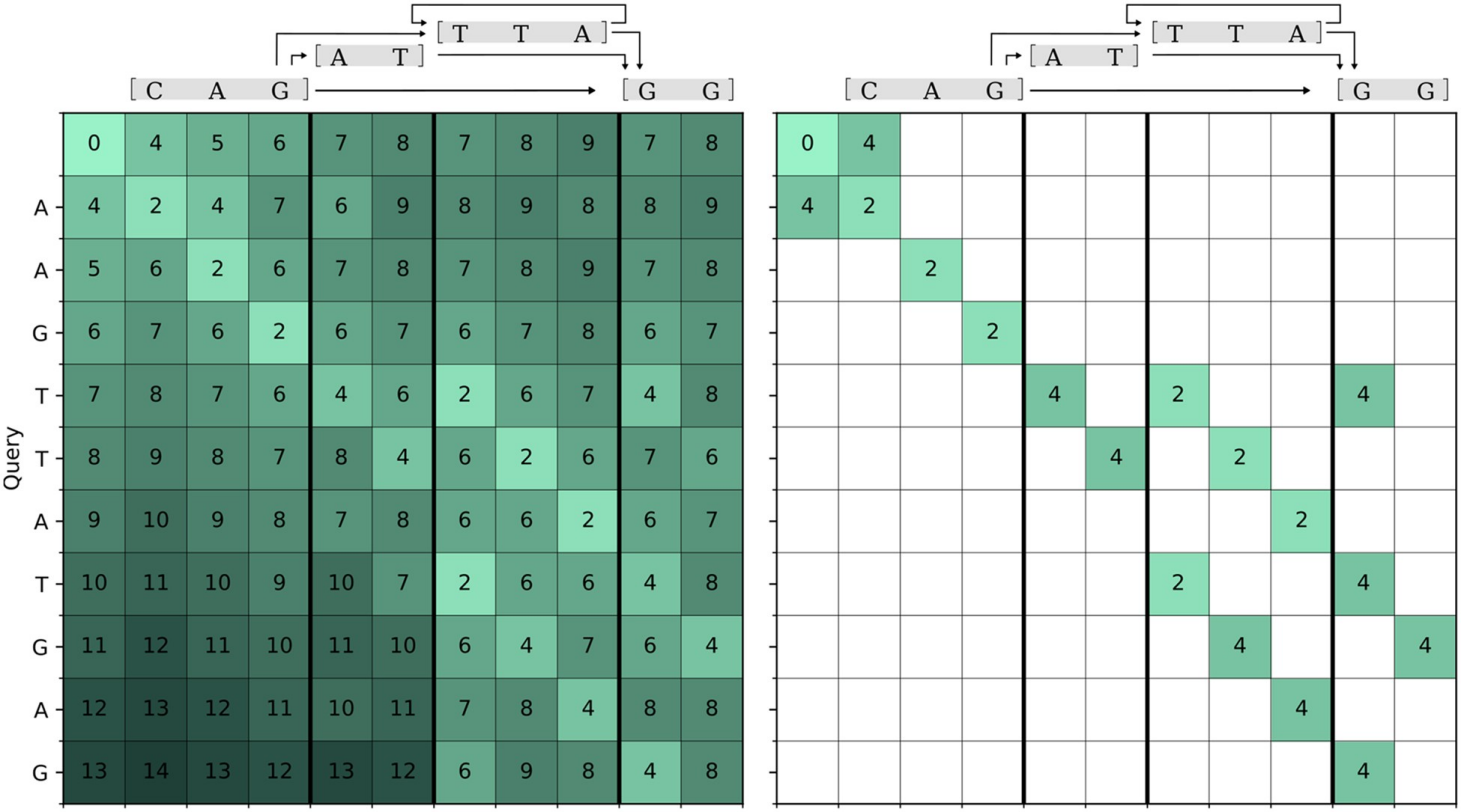
Comparison of the cells explored by traditional DP methods and the Theseus algorithm on a graph containing a cycle.

At every iteration, the algorithm maintains a set of active vertices $V_{\mathrm{act}}$, a set of active diagonals $K_{\mathrm{act}}$, and a set of invalidated diagonals $K_{\mathrm{inv}}$. We say that a vertex *v* is active if it has at least one active diagonal, where an active diagonal is defined as a diagonal in *v* that can be reached with score *s* and has not yet been invalidated. Thus, the active diagonals represent the positions within active vertices where the alignment can still progress. Limiting computation to this active set allows the algorithm to focus its effort on the regions of the graph that directly influence the alignment, improving both time and memory efficiency, as shown in Fig. 1. Each time a diagonal *k* reaches the end of a vertex, the diagonal is added to the set $K_{\mathrm{inv}}(v)$ of invalid diagonals in vertex *v*. An efficient implementation of this method, accounting for the implicit invalidation of diagonals due to gap propagation, is explained in Section 3.3.

The alignment process begins by setting the initial conditions. In the case of a global alignment starting at vertex $v_{0}$, it suffices to set ${\tilde{M}}_{v_{0},0}=0$ (score s=0, first wavefront). Initially, the set of active vertices is set to $V_{\mathrm{act}}=\{v_{0}\}$, the set of active diagonals is $K_{\mathrm{act}}(v_{0},{\tilde{M}}_{0})=0$, and the set of invalid diagonals $K_{\mathrm{inv}}$ is empty. The set of active vertices and diagonals is updated in each iteration due to jumps and the opening of new diagonals resulting from insertions and deletions, respectively. Then, for each score *s*, starting at s=0, Theseus performs the two core operations, *next* and *extend*, until the end condition is met at score $s^{*}$.

Algorithm 1 Theseus sequence-to-graph alignment algorithm.

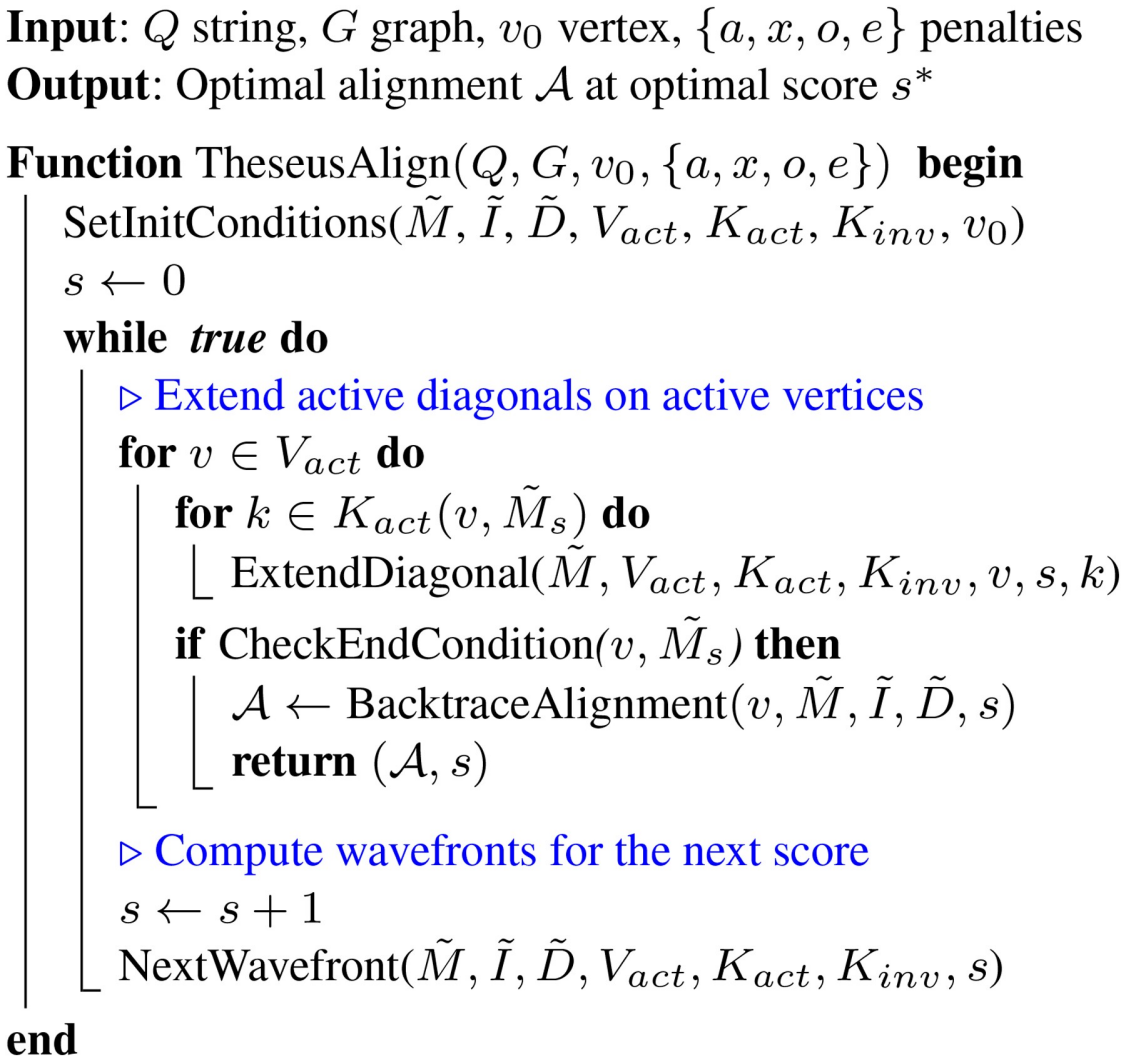

On each iteration of Algorithm 1, Theseus begins by extending each active diagonal (Algorithm 2). To that end, every active offset ${\tilde{M}}_{v,s,k}$ is greedily extended within its vertex *v* by computing the LCP between the suffixes $Q[{\tilde{M}}_{v,s,k},\ldots ,m]$ and $v[{\tilde{M}}_{v,s,k}+k,\ldots ,|v|]$. If the extension reaches the end of the vertex, that is, ${\tilde{M}}_{v,s,k}=|v|-k$, the diagonal *k* is invalidated in vertex *v*, and the algorithm proceeds to process every outgoing vertex *u* from *v*. Given a vertex *v*, we name $v_{\mathrm{out}}=\{u\in V$ | (v,u)∈E} the set of its outgoing vertices. For every $u\in v_{\mathrm{out}}$, *u* and *k* are marked as active (if they have not already been) and the value at ${\tilde{M}}_{u,s,k}$ diagonal *k* is updated. If the diagonal *k* in vertex *u* is not inactive, the extension continues within *u*. The algorithm repeats this procedure until it finds a mismatch. Most importantly, the algorithm makes no assumptions about the graph topology and correctly handles graphs containing cycles.

After extending all active diagonals, Theseus evaluates the end condition associated with the problem at hand. In the case of global alignment starting at vertex $v_{0}$, for instance, the algorithm satisfies the end condition the first time the query *Q* is completely processed. When Theseus meets this condition for the first time, it backtraces the path of an optimal alignment and returns it to the user, along with the optimal score $s=s^{*}$.

If the end condition is not met, Theseus computes the wavefronts with the next score s=s+1, as illustrated in Algorithm 3. For each active vertices *v*, the ${\tilde{I}}_{v,s}$, ${\tilde{D}}_{v,s}$, and ${\tilde{M}}_{v,s}$ wavefronts are computed based on the values of the corresponding wavefronts with a lower score, according to Equation (3). In particular, each time Theseus reaches the end of a vertex *v* with an insertion, it invalidates the corresponding diagonal and continues the alignment in its outgoing vertices $u\in v_{\mathrm{out}}$.

### 3.3 Diagonal invalidation strategy

According to Equation 3, the values at any vertex *v* may depend on wavefront values from adjacent vertices in the sequence graph. Once a diagonal *k* reaches the end of its associated vertex (i.e. ${\tilde{I}}_{v,s,k}=|v|-k$ or ${\tilde{M}}_{v,s,k}=|v|-k$, for some score *s*) its offsets propagate to all the outgoing vertices $u\in v_{\mathrm{out}}$. Thus, due to the score-ascending search of the solution space performed by Theseus, any later exploration of diagonal *k* in vertex *v* will be of a higher or equal score *s*, never leading to better-scoring paths. Therefore, Theseus can safely invalidate diagonal *k* in future wavefronts at vertex *v*, avoiding redundant work to compute suboptimal alignments.

Algorithm 2 ExtendDiagonal to advance diagonals.

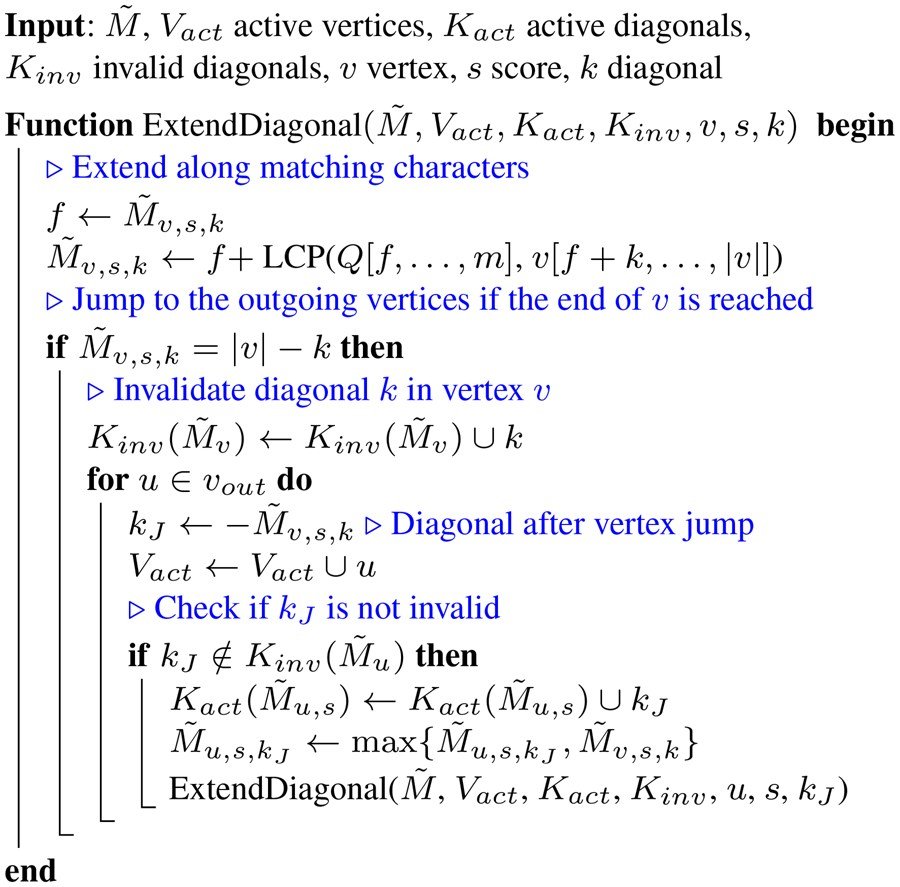

More in detail, a diagonal *k* can reach the end of a vertex *v* in two distinct situations, noted as *Jump* in Equation (3). In either of these two cases, Theseus sets the corresponding diagonal as invalid, either for $\tilde{M}$ or $\tilde{I}$. In addition, due to gap propagation on the outgoing vertices, Theseus implicitly invalidates neighboring diagonals every o+e or *e* scores. This is because the newly invalidated diagonals cannot produce better-scoring paths than those stemming from the associated diagonal on the outgoing vertices.

Theseus compactly represents the invalid diagonals as an ordered set of growing and non-overlapping segments of invalid diagonals. In particular, each segment consists of four values: the start and end of the range [min_diag,max_diag] of invalid diagonals, and the remaining scores rem_down and rem_up to invalidate adjacent diagonals due to gap propagation. It is important to note that, given a vertex *v*, the sets of invalid diagonals in $\tilde{M_{v}},\tilde{I_{v}}$ and $\tilde{D_{v}}$ are treated separately, as both $\tilde{I_{v}}$ and $\tilde{D_{v}}$ already incorporate the gap opening cost. Theseus initializes segments of invalid diagonals on *jumps*, either on $\tilde{M_{v}}$ or $\tilde{I_{v}}$. Then, at the end of each iteration on the main loop of Alg. 1, it decreases the scores remaining to expand the segment by one as well. If a remaining value reaches zero, it is set to the default *e* value, and the segment is expanded in the corresponding direction. Then, we sort and merge the updated set to keep it compact and non-overlapping.

This invalidation strategy is applicable to all types of directed graphs, including those with cycles. This is an important feature, as state-of-the-art diagonal invalidation strategies, such as the superbubble pruning implemented in POASTA, are limited to DAGs. Applying those strategies would hinder Theseus’s ability to align sequences when cycles are present in the reference. Overall, our method is designed to operate on general directed graphs, enabling it to accurately model and process repetitive regions in the reference pangenome.

### 3.4 Unified coordinate system for efficient sparse-wavefront processing

Due to the divergence of diagonals at outgoing vertices and their convergence at incoming ones, the wavefronts in Theseus are no longer inherently dense but present a sparse behavior. Consequently, we require sparse-data structures to represent them, along with novel methods for efficient processing.

Algorithm 3 NextWavefront to compute wavefronts of score *s*.

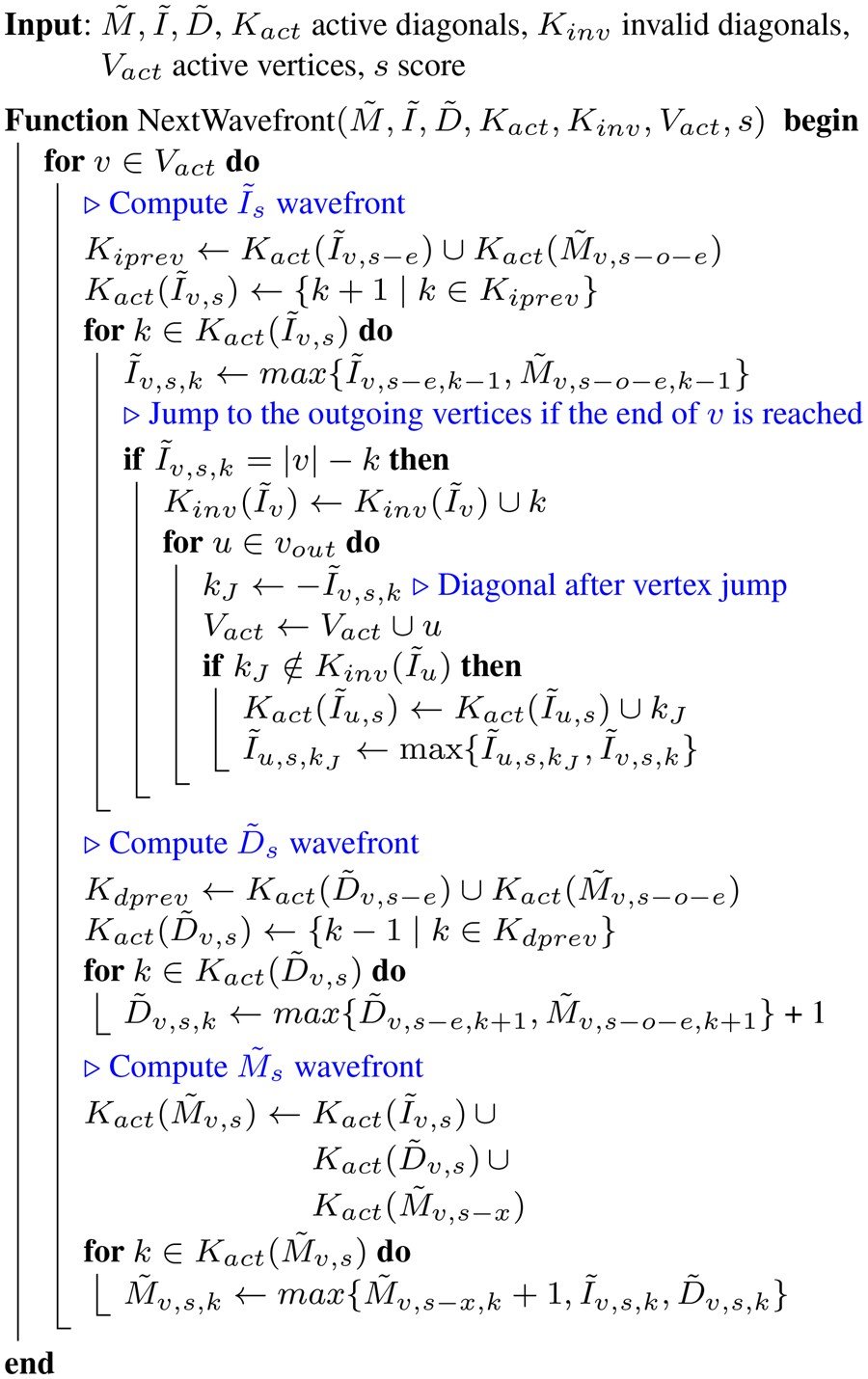

Theseus stores alignment information in sparse wavefronts. A sparse wavefront is a set of not necessarily ordered wavefront cells, where each of these cells stores the position of the cell in the corresponding DP matrix and additional information to perform the backtrace for the given alignment problem. However, processing sparse wavefronts introduces two challenges. First, because the cells are noncontiguous, their positions must be tracked individually. Second, the diagonals are often sparsely distributed, potentially unordered, and reused in later sparse wavefront computations, requiring a specialized strategy to process them efficiently.

First, to track sparse cells individually, Theseus proposes encoding each cell’s location using its diagonal (*k*), its offset (*off*) within that diagonal, and the vertex (*v*) where it resides.

Second, to efficiently combine sparse wavefronts across vertices and diagonals, we propose using a unified coordinate system (UCS). Recall m=|Q|, the length of the input sequence, and let $n={\text{max}}_{v}\{|v|\}$ be the maximum sequence length within the input sequence-graph *G*. According to Equation (3), the range of possible diagonals that Theseus can potentially explore is [−m,…,n]. Thus, we introduce an intermediate data structure that maps sparse wavefront data into a common coordinate system of size n+m+1, and then converts the result back to a sparse representation.

**Figure 2:**
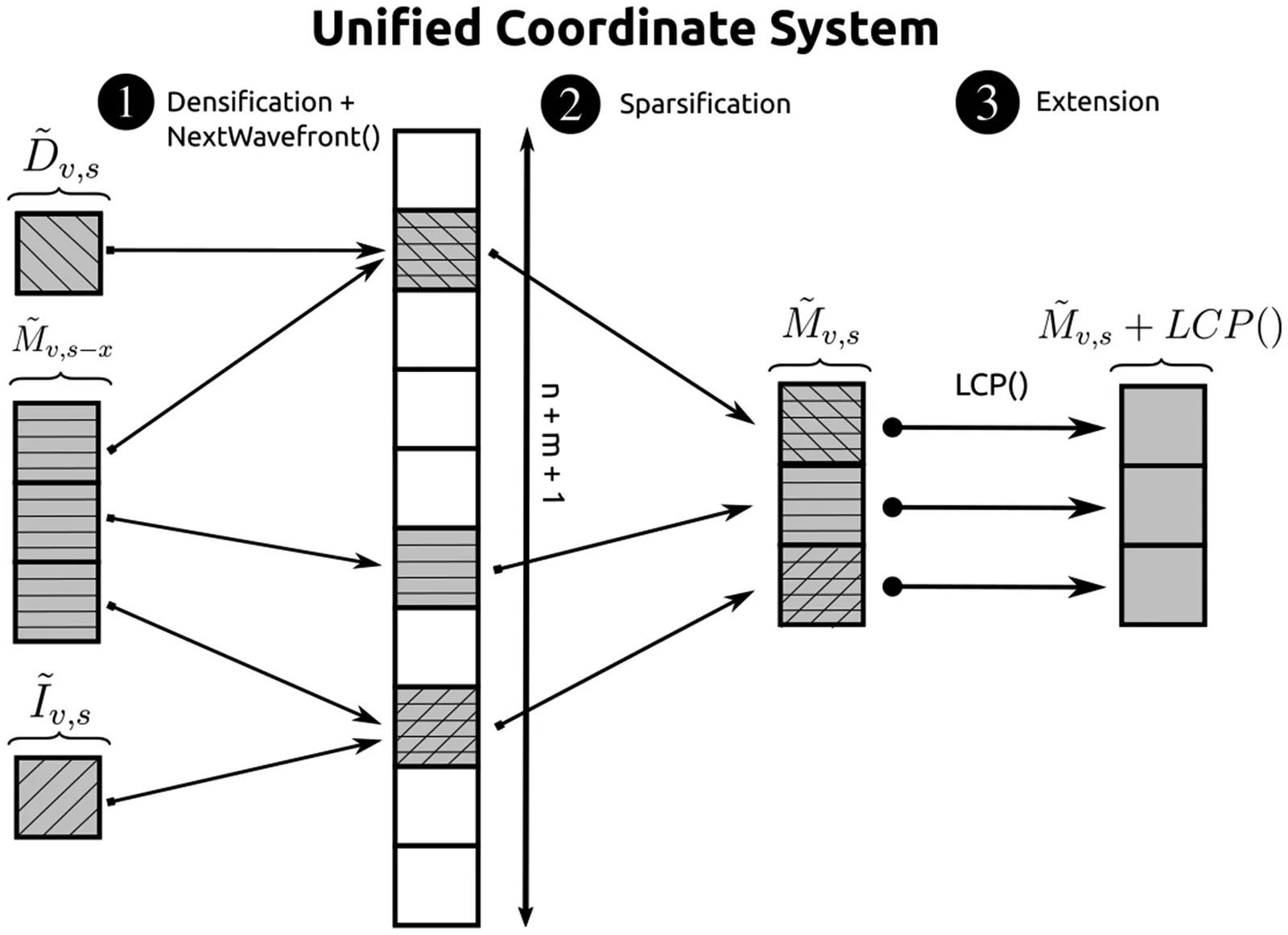
Example of how the Unified Coordinate System (UCS) combines previous sparse wavefronts.

Figure 2 shows an example of the UCS operation. First, during the *densification* step, each element of the preceding sparse wavefronts is mapped to its target position in the UCS according to Equation (3), implementing the NextWavefront operation. In this process, the recurrence is applied element-wise, so that each contribution updates its target diagonal. When multiple values map to the same UCS position, we resolve the collision by taking the maximum, as defined by the recurrence 3. Second, *sparsification* step, once all contributions have been inserted into the UCS, we store the updated diagonals to form the resulting sparse wavefront. Third, *extension* step, the ExtendDiagonal operation is executed on the resulting sparse wavefront. Finally, the UCS is prepared for a new iteration by resetting the modified diagonals to their default values. Figure 2 illustrates this three-step process.

### 3.5 Memory efficient sparse-wavefront backtrace

In sequence-to-graph alignment, the goal is often not only to compute the optimal alignment score, but also to recover the sequence of edit operations that transform *Q* into a sequence spelled by a path in *G*. In fact, backtracing alignments in a sequence graph requires storing additional information, such as the set and order of vertices visited (i.e. a path in the reference graph).

Backtracing the optimal alignment requires storing the computed results and, once the alignment is finished, reconstructing the set of alignment operations that led to the optimal alignment score. Because Theseus explores sparse wavefronts, each sparse wavefront cell stores an explicit pointer to the cell that generated it during the next or extend stages. After the alignment is completed, the backtracing algorithm traverses these linked pointers from the end cell back to the starting cell. Using this information Theseus is able to find the optimal alignment traceback, storing both the traversed path in the reference and the set of edit operations.

However, memory consumption is a key limiting factor in the scalability of any alignment algorithm. For this reason, Theseus takes inspiration from Singletrack (López-Villellas *et al.* 2026) to reduce the memory requirements of affine-gap alignment. The key observation of the Singletrack method is that, for sequence-to-sequence alignment, it is enough to store the data from the *M* matrix to recover the optimal alignment path, with negligible overhead during traceback.

We note that this observation also applies to sequence-to-graph alignment, with one additional requirement. Theseus can recover the optimal alignment path by storing the *M* matrix entries and a subset of the *I* matrix entries, with negligible overhead during traceback. The additional *I* entries are needed to reconstruct the vertex path followed by long insertions that span multiple graph vertices. Without this information, the backtrace algorithm can identify an insertion but not the sequence of vertices that it crosses.

We adapt the Singletrack method to the sequence-to-graph setting in Theseus, extending its memory-efficient backtrace strategy to handle vertex transitions while maintaining optimality. As a result, we reduce memory consumption by storing only the $\tilde{M}$ values and the subset of jumping $\tilde{I}$ values.

## 4 Results

We implemented Theseus as a C++ library, which is publicly available on Github. All datasets used for experimentation have been uploaded to Zenodo.

### 4.1 Experimental setup

We evaluate the performance of Theseus by comparing it with other state-of-the-art sequence-to-graph libraries and tools. First, in Section 4.2, we evaluate Theseus in the context of computing MSAs, comparing it with POA-based libraries such as SPOA (Vaser *et al.* 2017), abPOA (Gao *et al.* 2021), and POASTA (van Dijk *et al.* 2024). Second, in Section 4.3, we evaluate Theseus for pangenome read mapping, comparing its performance against the sequence-to-graph alignment stage of vg map (Garrison *et al.* 2018), along with the alignment kernels of SPOA, abPOA, and POASTA. Third, in Section 4.4, we evaluate Theseus as a sequence-to-cyclic graph aligner, comparing it with the alignment kernel of vg map. All experiments were executed single-threaded on an Intel Xeon Platinum 8480 CPU @3GHz node equipped with 256 GB of DRAM.

### 4.2 Evaluation on multiple sequence alignment (MSA)

**Figure 3:**
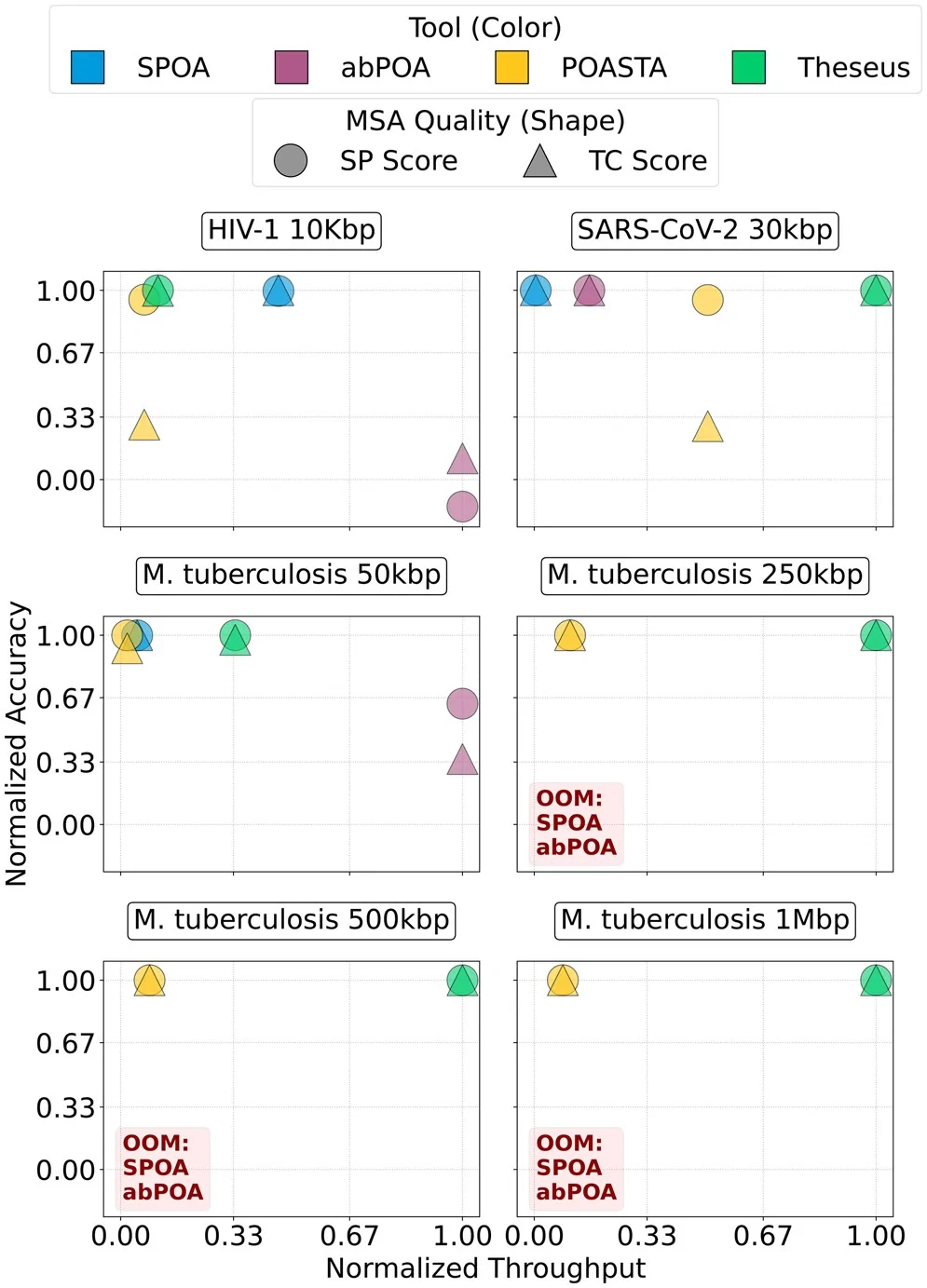
Plot combining normalized alignment throughput with normalized alignment accuracy. Values have been normalized with respect to the maximum for each dataset.

In this section, we evaluate Theseus’s sequence-to-graph alignment algorithm for computing MSAs against POA-based libraries such as SPOA, abPOA, and POASTA. It is important to note that all evaluated libraries produce optimal alignments, except for abPOA, which uses an adaptive banding strategy to accelerate computations, at the expense of producing suboptimal solutions. For the experiments, each aligner is provided with a set of genomic sequences that are added progressively to the POA graph until all sequences have been processed. Tables 1 and 2 measure the total time, and peak memory consumption spent aligning the sequences and updating the corresponding POA graph. Moreover, Fig. 3 shows alignment accuracy along with alignment throughput, contextualizing the tradeoff between accuracy and speed.

**Table 1:** Execution time (in seconds) of SPOA, abPOA, POASTA, and Theseus on different datasets for the problem of MSA.

Dataset	SPOA	abPOA	POASTA	Theseus
HIV-1 10 kb	26	12	174	110
SARS-CoV-2 30 kb	7664	205	65	33
*M. tuberculosis* 50 kb	2571	124	6512	370
*M. tuberculosis* 250 kb	oom	oom	8690	910
*M. tuberculosis* 500 kb	oom	oom	29 024	2453
*M. tuberculosis* 1 Mb	oom	oom	71 770	5996
Monkeypox 200 kb	oom	oom	timeout	7785

oom: execution exceeded available memory (out of memory).

timeout: execution exceeded 48 h.

**Table 2:** Peak memory consumption (in GB) of SPOA, abPOA, POASTA, and Theseus on different datasets for the problem of MSA.

Dataset	SPOA	abPOA	POASTA	Theseus
HIV-1 10 kb	4.5 GB	7.1 GB	0.4 GB	0.5 GB
SARS-CoV-2 30 kb	42.6 GB	13.5 GB	2.4 GB	0.8 GB
*M. tuberculosis* 50 kb	116.9 GB	66.0 GB	26.5 GB	53.1 GB
*M. tuberculosis* 250 kb	oom	oom	43.9 GB	52.2 GB
*M. tuberculosis* 500 kb	oom	oom	46.3 GB	52.7 GB
*M. tuberculosis* 1 Mb	oom	oom	59.0 GB	54.4 GB
Monkeypox 200 kb	oom	oom	timeout	204.9 GB

oom: execution exceeded available memory (out of memory).

timeout: execution exceeded 48 h.

For the evaluation, we follow a methodology similar to that used in the POASTA work. We selected 7 datasets of varying sequence lengths (10 kb–1 Mb): *HIV-1* 10 kb, *SARS-CoV-2* 30 kb, *M. tuberculosis* 50 kb, *M. tuberculosis* 250 kb, *M. tuberculosis* 500 kb, *M. tuberculosis* 1 Mb, and *Mokeypox* 200 kb. The *HIV-1* dataset contains 100 whole-genome assemblies of *HIV-1*. The *SARS-CoV-2* dataset contains 2732 GenBank-complete *SARS-CoV-2* genome assemblies, each approximately ∼30 kb in length. The *M. tuberculosis* 50 kb, 250 kb, 500 kb, and 1 Mb datasets are derived from the same set of 342 RefSeq-complete whole-genome assemblies of *Mycobacterium tuberculosis* genomes by extracting subsequences of different lengths, all starting at the *dnaG* gene. Finally, the *Monkeypox* dataset contains 100 RefSeq-complete whole genome assemblies of *Monkeypox’*s virus, each approximately 200 kb in length.

Table 1 shows the execution times of the MSA experiments. We observe that Theseus outperforms POASTA across all experiments, being between 1.6× and 17.6× faster. Compared to SPOA, Theseus is 4.2× slower in the *HIV-1* dataset, 6.9× faster in the *M. tuberculosis* 50 kb dataset, and 232.2× faster in the *SARS-CoV-2* dataset. Comparing our method to the heuristic abPOA, we observe that while abPOA is 3.0× faster on the *M. tuberculosis* 50 kb dataset and 9.2× faster in the *HIV-1* dataset, Theseus outperforms abPOA by 6.2× on the *SARS-CoV-2* dataset. More importantly, Theseus is guaranteed to always produce optimal alignments, whereas abPOA trades accuracy for speed through heuristic banding and only completes three experiments, running out of memory on datasets with sequences longer than 50 kb. Theseus is the only method that scales to longer sequences (i.e. 200 kb) while maintaining practical runtimes.

Additionally, Table 2 shows the peak memory consumption of the experiments comparing aligners. The results show that both SPOA and abPOA can only process the two smallest datasets, due to their large memory requirements, requiring 116.9GB and 66.0GB in the *M. tuberculosis* 50 kb problem, respectively. In contrast, POASTA and Theseus are able to process all datasets and exhibit similar memory usage that scales with the alignment score and problem size, although POASTA exceeds the 48 h runtime limit on the *Monkeypox* 200 kb-long dataset.

We complement the analysis with Fig. 3, which plots normalized alignment accuracy and normalized throughput. We measure alignment accuracy using the total column (TC) (Edgar 2004) and sum of pairs (SP) (Altschul 1989) scores. The TC score computes the number of totally conserved columns, and the SP computes the sum of the values of all pairs of positions in the MSA. Matches have a contribution of +1, mismatches of −1, and gaps of −1. We can observe that Theseus is consistently among the most accurate methods across all datasets, for both TC and SP scores. Moreover, Theseus presents the highest throughput on five out of the seven datasets. Even though abPOA is faster in the *HIV-1* and *M. Tuberculosis* 50 kb datasets, its speed comes at the cost of a lower accuracy.

### 4.3 Evaluation on read mapping

In this section, we evaluate Theseus’ performance for pangenome read-mapping. Pangenome read-mappers, such as vg map from the vg toolkit (Garrison *et al.* 2018), align sequencing reads to a variation graph by combining seeding against a reference graph and sequence-to-graph alignment. The mapping process in vg map starts by finding partial exact matches between the query and the graph, in the form of Maximal Exact Matches (MEMs). Then, these seeds are clustered to identify candidate alignment regions or subgraphs. Finally, vg performs affine-gap sequence-to-graph alignment of the query against those candidate subgraphs. Our evaluation focuses on the sequence-to-graph alignment stage, isolating it from other components of the pangenome read-mapping workflow to enable a fair and informative comparison.

To perform this analysis, we use vg map to extract a collection of sequence-to-graph alignment inputs, consisting of pangenome subgraphs induced from mapping different input sequences to a human pangenome reference (built on top of the GRCh37 reference genome and containing all variation from Phase 3 of the 1000 Genomes Project (1000 Genomes Project Consortium *et al.* 2015) with an allele frequency AF≥1%). For each input read mapped to the forward strand, we use vg map to identify clusters of Maximal Exact Matches (MEMs) and induce a candidate subgraph. As a result, we obtain a collection of sequence-to-graph alignment instances, each defined by an induced subgraph, an alignment sequence, and the starting mapping position in the graph. We modify the induced subgraph by adding an additional node pointing to the starting position to enable a fair comparison between the different methods. Then, we evaluate the performance of different aligners to compute the alignment of the input sequence to the induced subgraph.

**Figure 4:**
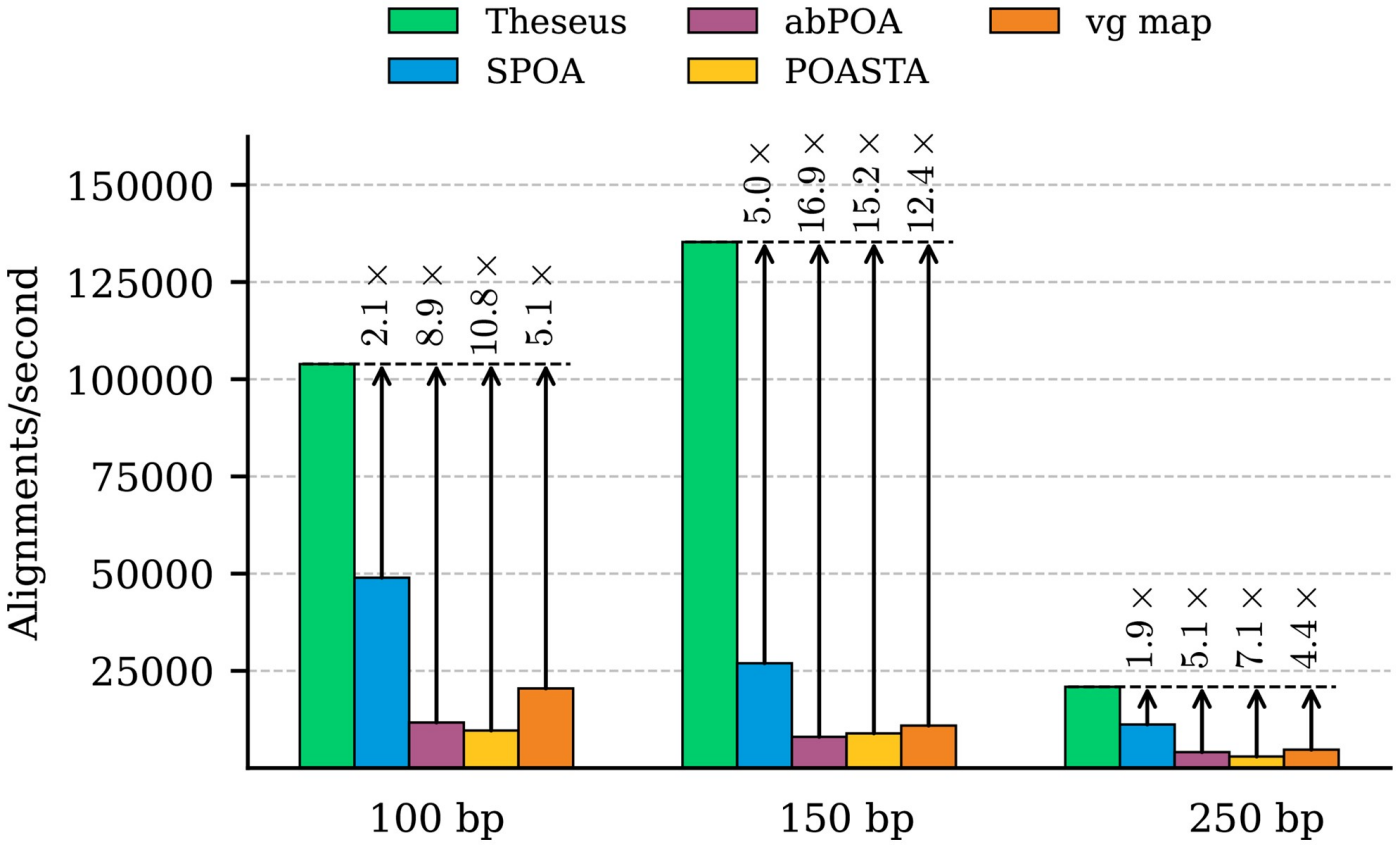
Alignment throughput of Theseus compared to other aligners on three datasets of 100 bp, 150 bp, and 250 bp.

Figure 4 evaluates the alignment kernels of vg map (Garrison *et al.* 2018), SPOA, abPOA, POASTA, and Theseus on three short read datasets of lengths 100 bp, 150 bp, and 250 bp. The Illumina 100 bp dataset comes from individual HG00096 of the 1000 Genomes Project and the other two were extracted from the Genome in a Bottle project (Zook *et al.* 2016). We measure alignment throughput by each sequence-to-graph aligner across the selected datasets.

We observe that Theseus is between 1.9× and 16.9× faster than the other alignment kernels. Note that the performance of Theseus is sensitive to the alignment difficulty (i.e. alignment score) of the input sequences across the different datasets and sequencing technologies.

### 4.4 Evaluation on cyclic graphs

Cyclic reference graphs arise naturally when modeling repetitive regions of a pangenome, such as tandem repeats or segmental duplications. Aligning to such graphs is an important capability.

However, POA-based methods operate on partial-order graphs, which are directed acyclic graphs (DAGs), and therefore cannot directly represent or align against cyclic reference graphs. A common way to use POA-based aligners on cyclic references is graph unfolding. Graph unfolding replaces cyclic regions with an acyclic graph that explicitly represents repeated traversals of those cycles. This is done by duplicating the vertices and edges within each cyclic component as many times as needed to represent all cycle traversals compatible with the query length. The resulting unfolded graph may be substantially larger than the original cyclic graph (Kavya *et al.* 2019). This is the strategy adopted by vg to align against cyclic references, since vg’s internal POA-based sequence-to-graph kernel does not natively support cyclic graphs.

Unlike POA-based aligners, Theseus directly supports aligning sequences to graphs that contain cycles. It does so on the original cyclic graph, without unfolding and without modifying the reference graph. This allows Theseus to preserve the compact cyclic representation while avoiding the additional graph growth introduced by unfolding.

In this section, we evaluate Theseus on synthetic cyclic graphs and compare it with vg, which requires unfolding to process cyclic references. To this end, we generate synthetic cyclic graphs with a controlled structure. For that, we create backbone graphs with N∈{10,100,1000} nodes, with node sequences of average length 50 bp, and connect each node to its successor to ensure graph connectivity. Then, we add a bounded number of random edges per node. These random edges can connect to nearby forward nodes or to previously created nodes, introducing cycles into the graph. For each graph size, we generate queries of 100, 150, and 250 bp. Then, we align the queries using Theseus on the original cyclic graphs and vg on the corresponding unfolded graphs to measure alignment throughput.

**Figure 5:**
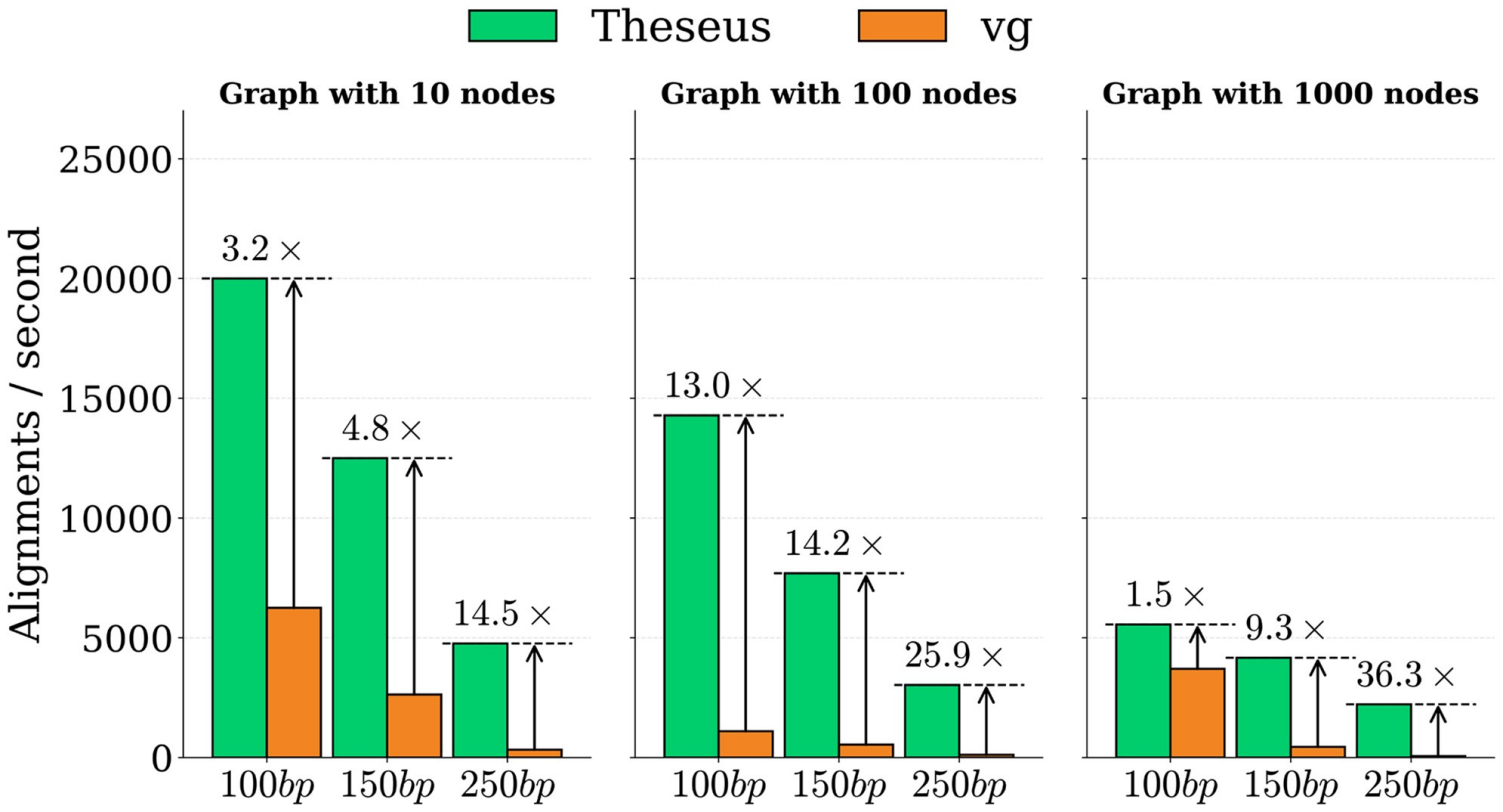
Alignment throughout of Theseus compared to the sequence-to-graph alignment kernel of vg map aligning reads to cyclic reference graphs.

Figure 5 compares the alignment throughput of Theseus on the original cyclic graphs with the alignment throughput of vg map’s alignment kernel. We observe that Theseus is between 1.5× and 36.3× faster than vg, with higher speedups for longer reads.

## 5 Discussion

In this work, we present Theseus, a fast, scalable, and affine-gap sequence-to-graph aligner. Theseus exploits the expected high similarity between genomic sequences to accelerate alignment and reduce its memory footprint. Compared to other alternatives, Theseus naturally supports cycles, making it adequate to handle all types of directed graphs.

We introduce several algorithmic innovations. First, we extend the affine-gap recurrence equations from the original algorithm to work with graph-based sequence references. Second, we implement a diagonal invalidation strategy that tracks previously processed and implicitly explored diagonals to avoid redundant computation. Third, we introduce the notions of sparse wavefronts and a Unified Coordinate System to handle the non-contiguous data arising from graph alignment. Finally, we introduce a memory-efficient backtrace algorithm, based on the recently published Singletrack (López-Villellas *et al.* 2026), tailored to the sparsity of sequence-to-graph alignment.

We evaluate Theseus across two different use cases to demonstrate its potential as a general sequence-to-graph aligner. For the MSA problem, Theseus is 1.6× to 17.6× faster than POASTA, and 7.3× faster, on average, than SPOA. Compared to abPOA, a heuristic aligner, Theseus is always either faster or more accurate. We also note that Theseus is the only tool that scales to larger problems while maintaining practical runtimes. For pangenome read mapping, we compare Theseus with vg map, SPOA, POASTA, and abPOA on short reads. We observe that Theseus is between 1.9× and 16.9× faster than the other aligners. Moreover, we compare Theseus against vg when aligning sequences to synthetic cyclic graphs. We observe that Theseus is 1.5× to 36.3× faster than vg.

Notwithstanding, the Theseus alignment algorithm has some limitations. Its runtime and memory scale with the optimal alignment score rather than with the input size. When sequences are highly similar, as is typical of pangenome graphs and conspecific MSA, the score is small relative to the sequence lengths, and Theseus achieves substantial speedups and memory reductions. As similarity decreases, the alignment score increases, and Theseus must explore a larger portion of the solution space to find the optimal alignment, reducing its advantage over other methods. For instance, on the most highly variable dataset (*HIV-1*), SPOA and the heuristic method abPOA are faster than Theseus.

Also note that SPOA and abPOA implementations rely on SIMD vectorization to accelerate their DP computations, whereas the present version of Theseus does not heavily exploit SIMD instructions. Likewise, the Theseus’ implementation is currently single-threaded and does not parallelize the alignment of a single query across cores. Adapting the sparse-wavefront operations for SIMD execution and exploring intra-alignment thread parallelism are promising directions for future work that could further improve Theseus’s performance.

Overall, Theseus bridges the gap between optimality and scalability in sequence-to-graph alignment. By combining output-sensitive exploration with sparse wavefront processing, it enables affine-gap alignment on large and cyclic graphs at a practical cost. As a result, we expect Theseus to provide a suitable building block for next-generation MSA pipelines and scalable pangenome analysis as reference graphs and datasets continue to grow in the future.

## Supplementary Material

btag561_Supplementary_Data

## Data Availability

The datasets used for the experimental evaluation in this article are available in Zenodo at https://doi.org/10.5281/zenodo.21132331.

## References

[btag561-B1] 1000 Genomes Project Consortium, Auton A, Brooks LD et al A global reference for human genetic variation. Nature 2015;526:68. 26432245 10.1038/nature15393 PMC4750478

[btag561-B2] Aguado-Puig Q , Doblas M, Matzoros C et al WFA-GPU: gap-affine pairwise read-alignment using GPUs. Bioinformatics 2023;39:btad701. 37975878 10.1093/bioinformatics/btad701 PMC10697739

[btag561-B3] Alonso-Marín A , Fernandez I, Aguado-Puig Q et al BIMSA: accelerating long sequence alignment using processing-in-memory. Bioinformatics 2024;40:btae631. 39432682 10.1093/bioinformatics/btae631 PMC11576351

[btag561-B4] Altschul SF. Gap costs for multiple sequence alignment. J Theor Biol 1989;138:297–309. 2593679 10.1016/s0022-5193(89)80196-1

[btag561-B5] Chin C-S , Wagner J, Zeng Q et al A diploid assembly-based benchmark for variants in the major histocompatibility complex. Nat Commun 2020;11:4794. 32963235 10.1038/s41467-020-18564-9 PMC7508831

[btag561-B6] Compeau PE , Pevzner PA, Tesler G. How to apply de bruijn graphs to genome assembly. Nat Biotechnol 2011;29:987–91. 22068540 10.1038/nbt.2023 PMC5531759

[btag561-B7] Computational Pan-Genomics Consortium. Computational pan-genomics: status, promises and challenges. Brief Bioinform 2018;19:118–35. 27769991 10.1093/bib/bbw089 PMC5862344

[btag561-B8] Darby CA , Gaddipati R, Schatz MC et al Vargas: heuristic-free alignment for assessing linear and graph read aligners. Bioinformatics 2020;36:3712–8. 32321164 10.1093/bioinformatics/btaa265 PMC7320598

[btag561-B9] Doblas M , Lostes-Cazorla O, Aguado-Puig Q et al Gmx: Instruction set extensions for fast, scalable, and efficient genome sequence alignment. In: *Proceedings of the 56th Annual IEEE/ACM International Symposium on Microarchitecture, Toronto, ON, Canada*. New York, NY, United States: Association for Computing Machinery, 2023, 1466–80.

[btag561-B10] Doblas M , Lostes-Cazorla O, Aguado-Puig Q et al Quicked: high-performance exact sequence alignment based on bound-and-align. Bioinformatics 2025a;41:btaf112. 40080714 10.1093/bioinformatics/btaf112 PMC11937955

[btag561-B11] Doblas M , Shih PJ, Lostes-Cazorla O et al Smx: heterogeneous architecture for universal sequence alignment acceleration. In: *Proceedings of the 58th IEEE/ACM International Symposium on Microarchitecture, Seoul, Korea*. New York, NY, United States: Association for Computing Machinery, 2025b, 1656–71.

[btag561-B12] Edgar RC. Muscle: multiple sequence alignment with high accuracy and high throughput. Nucleic Acids Res 2004;32:1792–7. 15034147 10.1093/nar/gkh340 PMC390337

[btag561-B13] Eizenga JM , Novak AM, Sibbesen JA et al Pangenome graphs. Annu Rev Genomics Hum Genet 2020;21:139–62. 32453966 10.1146/annurev-genom-120219-080406 PMC8006571

[btag561-B14] Gao Y , Liu Y, Ma Y et al Abpoa: an simd-based c library for fast partial order alignment using adaptive band. Bioinformatics 2021;37:2209–11. 33165528 10.1093/bioinformatics/btaa963

[btag561-B15] Garrison E , Sirén J, Novak AM et al Variation graph toolkit improves read mapping by representing genetic variation in the reference. Nat Biotechnol 2018;36:875–9. 30125266 10.1038/nbt.4227 PMC6126949

[btag561-B16] Goodwin S , McPherson JD, McCombie WR. Coming of age: ten years of next-generation sequencing technologies. Nat Rev Genet 2016;17:333–51. 27184599 10.1038/nrg.2016.49 PMC10373632

[btag561-B17] Gotoh O. An improved algorithm for matching biological sequences. J Mol Biol 1982;162:705–8. 7166760 10.1016/0022-2836(82)90398-9

[btag561-B18] Gotoh O. Multiple sequence alignment: algorithms and applications. Adv Biophys 1999;36:159–206. 10463075 10.1016/s0065-227x(99)80007-0

[btag561-B19] Haghi A , Marco-Sola S, Alvarez L et al WFA-FPGA: an efficient accelerator of the wavefront algorithm for short and long read genomics alignment. Future Generation Computer Systems 2023;149:39–58.

[btag561-B20] Hart PE , Nilsson NJ, Raphael B. A formal basis for the heuristic determination of minimum cost paths. IEEE Trans Syst Sci Cyber 1968;4:100–7.

[btag561-B21] Ivanov P , Bichsel B, Mustafa H et al Astarix: fast and optimal sequence-to-graph alignment. In: *International Conference on Research in Computational Molecular Biology, Padua, Italy*. Cham, Switzerland and Springer-Verlag Berlin, Heidelberg: Springer, 2020, 104–19.

[btag561-B22] Jain C , Zhang H, Gao Y et al On the complexity of sequence-to-graph alignment. J Comput Biol 2020;27:640–54.

[btag561-B23] Kavya VNS , Tayal K, Srinivasan R et al Sequence alignment on directed graphs. J Comput Biol 2019;26:53–67. 30204489 10.1089/cmb.2017.0264

[btag561-B24] Lee C , Grasso C, Sharlow MF. Multiple sequence alignment using partial order graphs. Bioinformatics 2002;18:452–64. 11934745 10.1093/bioinformatics/18.3.452

[btag561-B25] Li H , Feng X, Chu C. The design and construction of reference pangenome graphs with minigraph. Genome Biol 2020;21:265. 33066802 10.1186/s13059-020-02168-z PMC7568353

[btag561-B26] Liao W-W , Asri M, Ebler J et al A draft human pangenome reference. Nature 2023;617:312–24. 37165242 10.1038/s41586-023-05896-x PMC10172123

[btag561-B27] López-Villellas L , Pineda-Sánchez E, Badouh A et al Risc-v for genome data analysis: opportunities and challenges. In: *2023 38th Conference on Design of Circuits and Integrated Systems (DCIS), Málaga, Spain*. New York City, NY, USA: IEEE, 2023, 1–6.

[btag561-B28] López-Villellas L , Langarita-Benítez R, Badouh A et al Genarchbench: a genomics benchmark suite for arm hpc processors. Future Generation Computer Systems 2024;157:313–29.

[btag561-B29] López-Villellas L , Iñiguez C, Jiménez-Blanco A et al Singletrack: an algorithm for improving memory consumption and performance of gap-affine sequence alignment. Bioinformatics 2026;42:btag183. 10.1093/bioinformatics/btag183 PMC13171181 41981735

[btag561-B30] Marco-Sola S , Ribeca P. Efficient alignment of illumina-like high-throughput sequencing reads with the genomic multi-tool (GEM) mapper. Curr Protoc Bioinformatics 2015;50:11–3. 10.1002/0471250953.bi1113s50 26094690

[btag561-B31] Marco-Sola S , Sammeth M, Guigó R et al The gem mapper: fast, accurate and versatile alignment by filtration. Nat Methods 2012;9:1185–8. 23103880 10.1038/nmeth.2221

[btag561-B32] Marco-Sola S , Moure JC, Moreto M et al Fast gap-affine pairwise alignment using the wavefront algorithm. Bioinformatics 2021;37:456–63. 32915952 10.1093/bioinformatics/btaa777 PMC8355039

[btag561-B33] Marco-Sola S , Eizenga JM, Guarracino A et al Optimal gap-affine alignment in o (s) space. Bioinformatics 2023;39:btad074. 36749013 10.1093/bioinformatics/btad074 PMC9940620

[btag561-B34] Miga KH , Wang T. The need for a human pangenome reference sequence. Annu Rev Genomics Hum Genet 2021;22:81–102. 33929893 10.1146/annurev-genom-120120-081921 PMC8410644

[btag561-B35] Miller JR , Koren S, Sutton G. Assembly algorithms for next-generation sequencing data. Genomics 2010;95:315–27. 20211242 10.1016/j.ygeno.2010.03.001 PMC2874646

[btag561-B36] Myers G. A fast bit-vector algorithm for approximate string matching based on dynamic programming. J ACM 1999;46:395–415.

[btag561-B37] Navarro G. Improved approximate pattern matching on hypertext. Theor Comput Sci 2000;237:455–63.

[btag561-B38] Needleman SB , Wunsch CD. A general method applicable to the search for similarities in the amino acid sequence of two proteins. J Mol Biol 1970;48:443–53. 5420325 10.1016/0022-2836(70)90057-4

[btag561-B39] Rautiainen M , Marschall T. Graphaligner: rapid and versatile sequence-to-graph alignment. Genome Biol 2020;21:253. 32972461 10.1186/s13059-020-02157-2 PMC7513500

[btag561-B40] Rautiainen M , Mäkinen V, Marschall T. Bit-parallel sequence-to-graph alignment. Bioinformatics 2019;35:3599–607. 30851095 10.1093/bioinformatics/btz162 PMC6761980

[btag561-B41] Tettelin H , Riley D, Cattuto C et al Comparative genomics: the bacterial pan-genome. Curr Opin Microbiol 2008;11:472–7. 19086349 10.1016/j.mib.2008.09.006

[btag561-B42] Van Dijk LR , Manson AL, Earl AM et al Fast and exact gap-affine partial order alignment with POASTA. *Bioinformatics* 2024;**41:btae757. **https://doi.org/10.1093/bioinformatics/btae757 10.1093/bioinformatics/btae757 PMC11755094 39752324

[btag561-B43] Vaser R , Sović I, Nagarajan N et al Fast and accurate de novo genome assembly from long uncorrected reads. Genome Res 2017;27:737–46. 28100585 10.1101/gr.214270.116 PMC5411768

[btag561-B44] Zhang B , Zhu W, Diao S et al The poplar pangenome provides insights into the evolutionary history of the genus. Commun Biol 2019;2:215. 31240253 10.1038/s42003-019-0474-7 PMC6581948

[btag561-B45] Zook JM , Catoe D, McDaniel J et al Extensive sequencing of seven human genomes to characterize benchmark reference materials. Sci Data 2016;3:160025–6. 27271295 10.1038/sdata.2016.25 PMC4896128

