# Supplementary material for: Theseus: fast and optimal affine-gap sequence-to-graph alignment
Source: Bioinformatics. 2026 Jul 27;42(9):btag561. doi: 10.1093/bioinformatics/btag561 (PMC13544969; doi:10.1093/bioinformatics/btag561)
Supplement: Supplementary file 1 [file btag561_supplementary_data.pdf]

Theseus: Fast and Optimal Affine-Gap Sequence-to-Graph Alignment

Supplementary Material

Albert Jiménez-Blanco[1,2], Lorién López-Villellas[4], Juan Carlos Moure[3], Miquel Moretó[2,5], and Santiago Marco-Sola[1,2]

[1]Department of Computer Science, Universitat Politècnica de Catalunya, Barcelona, Spain.
[2]Computer Sciences Department, Barcelona Supercomputing Center, Barcelona, Spain.
[3]Department of Computer Architecture and Operative Systems, Universitat Autònoma de Barcelona, Barcelona, Spain.
[4]Department of Computer Science and Systems Engineering, University of Zaragoza, Spain.
[5]Department of Computer Architecture, Universitat Politècnica de Catalunya, Barcelona, Spain.

Summary

This supplementary material provides additional details and technical content that complement the main manuscript, including:

- An extended analysis of the accuracy of the MSA's of the compared POA aligners.
- An in-depth exploration of Theseus' capacity to natively handle cyclic graphs, compared to vg's graph unfolding strategy.
- An analysis on the effect on memory usage of applying Singletrack's ideas to Theseus.
- A comparison against MAFFT, a Multiple Sequence Aligner based on the Fast Fourier Transform.

1 Extended Accuracy Analysis

Even though two distinct POA aligners are optimal, this does not guarantee that the resulting POA graph is the same. This happens because distinct POA aligners can have different tie-breaking decisions on the backtrace stage and might implement different stopping conditions. The difference in the final POA graph is even bigger when comparing against heuristic aligners, such as abPOA, which trade off optimality for speed.

To evaluate the accuracy of the POA aligners that we compare in this work, we compute the total column (TC) and sum-of-pairs (SP) scores. For the SP scores, we set the contribution of matches to be +1 and that of mismatches and gaps to be -1. Therefore, we compute the SP scores as the sum of the contributions of all substitutions and gaps. We exclude the analysis of the Monkeypox dataset, as only Theseus is able to complete it.

In Table S1, we see that Theseus consistently presents high TC scores across all datasets. Similarly, SPOA achieves the maximum values across the three datasets it can process.

Table S1: Total-Column (TC) scores for the MSA evaluation. Experiments that ran out of memory are marked as oom.

DATASET	SPOA	abPOA	POASTA	Theseus
HIV-1 10Kbp	2862	320	830	2853
SARS-CoV-2 30kbp	23419	23402	6604	23417
M. tuberculosis 50kbp	33198	11480	30904	32386
M. tuberculosis 250kbp	*oom*	*oom*	215634	215644
M. tuberculosis 500kbp	*oom*	*oom*	428658	428558
M. tuberculosis 1Mbp	*oom*	*oom*	872157	872074

The other tools present at least one dataset with very low TC scores. AbPOA in the HIV-1 and M. tuberculosis 50kbp datasets, and POASTA on the HIV-1 and SARS-CoV-2 datasets.

Table S2: Sum-of-Pairs (SP) scores for the MSA evaluation. Experiments that ran out of memory are marked as oom.

DATASET	SPOA	abPOA	POASTA	Theseus
HIV-1 10Kbp	27993502	-4018311	26709094	28103366
SARS-CoV-2 30kbp	110220375889	110244181637	104607811726	110236629116
M. tuberculosis 50kbp	2770099166	1769809325	2770008958	2772274389
M. tuberculosis 250kbp	*oom*	*oom*	14184334809	14189860665
M. tuberculosis 500kbp	*oom*	*oom*	28570475484	28562290016
M. tuberculosis 1Mbp	*oom*	*oom*	57363381785	57365784080

Similarly, Table S2 presents the Sum-of-Pairs (SP) score. We observe that all algorithms, except abPOA, consistently achieve high SP values. It should be noted that abPOA is a heuristic method that improves speed by relaxing optimality guarantees, potentially reducing TC and SP scores.

2 Comparison of Theseus' Native Handling of Cyclic-Graphs Against vg's Graph-Unfolding Strategy

Like most existing sequence-to-graph alignment tools, vg does not natively support aligning a query sequence Q against a reference graph G that contains cycles, as it can only align sequences to directed acyclic graphs (DAGs). However, there is a strategy to implicitly support cyclic graphs with an algorithm that doesn't natively support them: graph unfolding. The graph unfolding strategy replaces cyclic regions with an acyclic graph that explicitly enumerates the possible traversals of those cycles. This is done by duplicating the vertices and edges within the cyclic component as many times as needed to achieve the query length. The resulting graph can then be processed by a DAG aligner, but it may be substantially larger than the original cyclic graph (Kavya *et al.*, 2019).

In this experiment, we evaluate Theseus on synthetic cyclic graphs and compare it with vg, which requires unfolding to process cyclic references. To this end, we generate synthetic cyclic graphs with a controlled structure. For that, we create backbone graphs

with N $\in \{10, 100, 1000\}$ nodes, with node sequences of average length 50 bp, and connect each node to its successor to ensure graph connectivity. Then, we add a bounded number of random edges per node. These random edges can connect to nearby forward nodes or to previously created nodes, introducing cycles into the graph. For each graph size, we generate queries of length 100, 150, and 250 bp. Then, we align the queries using Theseus on the original cyclic graphs and vg on the corresponding unfolded graphs, and measure alignment throughput.

Table S3 summarizes the results of this experiment. We see that Theseus has a higher alignment throughput than vg across all datasets, with speedups between 1.5× and 36.3×. It is also interesting to note that graph unfolding has a significant effect on graph size, which directly impacts vg's performance. On average, the unfolded graphs have 2.9× more nodes than the original graphs.

Table S3: Execution time of Theseus and the alignment kernel of vg map aligning reads to cyclic reference graphs. We also evaluate the increase in the number of nodes caused by graph unfolding.

Graph size	Read length	Node increase	vg's Time	Theseus' Time
10 nodes	100 bp	2.4×	1.6 s	0.5 s
	150 bp	2.8×	3.8 s	0.8 s
	250 bp	7.8×	30.5 s	2.1 s
100 nodes	100 bp	4.4×	9.1 s	0.7 s
	150 bp	3.5×	18.5 s	1.3 s
	250 bp	4.5×	85.4 s	3.3 s
1000 nodes	100 bp	1.0×	2.7 s	1.8 s
	150 bp	1.8×	22.4 s	2.4 s
	250 bp	2.5×	163.5 s	4.5 s

3 Memory Impact of Singletrack in Theseus

We have evaluated the effect of using Singletrack's backtrace strategy in four distinct datasets from the MSA evaluation. Table S4 summarizes memory consumption in GB of a version of Theseus using Singletrack and another without it. We have performed this analysis in four datasets: SARS-CoV-2 30kbp, M. tuberculosis 50kbp, M. tuberculosis 250kbp, and M. tuberculosis 500kbp. We observe that Theseus with Singletrack uses between 1.5× and 2.5× less memory than the version not implementing Singletrack.

4 Comparison Against MAFFT

MAFFT (Katoh *et al.*, 2002) is a multiple sequence aligner that detects homologous regions using the fast Fourier transform (FFT). It implements an iterative refinement method that progressively aligns the input sequences according to the branching order of a given guide tree.

In consequence, MAFFT is not a POA-based MSA method and does not rely on a sequence-to-graph alignment kernel. Thus, it provides a useful MSA-level comparison,

Dataset	Theseus without ST	Theseus with ST
SARS-CoV-2 30kbp	1.2	0.8
M. tuberculosis 50kbp	104.0	53.1
M. tuberculosis 250kbp	127.5	52.2
M. tuberculosis 500kbp	129.3	52.7

Table S4: Peak memory consumption (in GB) of Theseus using Singletrack's ideas and Theseus without using them.

but it does not directly evaluate the kernel accelerated by Theseus. For completeness, we have evaluated MAFFT on all MSA datasets evaluated in the manuscript, using different thread counts. We summarize the results in Tables S5 and S6.

Table S5: Execution Time (in seconds) of MAFFT with 1, 4, 16, and 32 threads, and Theseus on different datasets for the problem of MSA.

Dataset	MAFFT 1	MAFFT 4	MAFFT 16	MAFFT 32	Theseus
HIV-1 10Kbp	12	9	9	9	110
SARS-CoV-2 30kbp	899	253	102	89	33
M. tuberculosis 50kbp	1991	988	768	768	370
M. tuberculosis 250kbp	12642	5781	4510	4510	910
M. tuberculosis 500kbp	44247	23555	17404	17285	2453
M. tuberculosis 1Mbp	60243	38535	35118	35099	5996
Monkeypox 200Kbp	2666	1547	1417	1425	7785

Table S6: Peak memory consumption (in GB) of MAFFT with 1, 4, 16, and 32 threads, and Theseus on different datasets for the problem of MSA.

Dataset	MAFFT 1	MAFFT 4	MAFFT 16	MAFFT 32	Theseus
HIV-1 10Kbp	0.2 GB	0.5 GB	0.9 GB	0.9 GB	0.5 GB
SARS-CoV-2 30kbp	0.7 GB	1.2 GB	2.4 GB	2.0 GB	0.8 GB
M. tuberculosis 50kbp	0.3 GB	0.7 GB	1.0 GB	0.8 GB	53.1 GB
M. tuberculosis 250kbp	1.0 GB	3.0 GB	4.1 GB	4.1 GB	52.2 GB
M. tuberculosis 500kbp	2.1 GB	8.4 GB	11.9 GB	13.1 GB	52.7 GB
M. tuberculosis 1Mbp	4.1 GB	17.2 GB	17.8 GB	17.2 GB	54.4 GB
Monkeypox 200Kbp	0.3 GB	0.9 GB	1.7 GB	1.7 GB	204.9 GB

In Table S5, measuring execution time, we observe that Theseus is the fastest-running algorithm across all datasets except for the Monkeypox 200Kbp and the HIV-1 10Kbp datasets. Compared to MAFFT using 1 thread, Theseus is 2.9× slower on the Monkeypox dataset, 9.2× slower on the HIV-1 dataset, and between 5.4× and 27.2× faster on the other 5 datasets. As a result, Theseus is 3.9× faster on average than MAFFT using 1 thread. When comparing Theseus using 1 thread to MAFFT using various thread counts, we see the same pattern: Theseus is still the fastest-running algorithm in five out of the seven evaluated datasets, even when executing MAFFT with 32 threads.

Table S6 measures peak memory usage. Results show that MAFFT with 1 has the lowest memory consumption across all datasets. Theseus presents similar memory

requirements for the HIV-1 and SARS-CoV-2 datasets, but its memory consumption is much higher across the remaining datasets. This happens because Theseus, as POASTA, is an output-sensitive algorithm, and both runtime and memory consumption depend on the alignment score. In datasets 3-7, there is at least one highly erroneous sequence, which significantly increases peak memory consumption. When compared to the other POA aligners, however, Theseus' memory consumption is competitive across all datasets. SPOA and abPOA have much higher memory requirements and run out of memory on 4 of the 7 evaluated datasets, while POASTA suffers from the same memory bottleneck when very erroneous sequences are present.

References

Katoh, K., Misawa, K., Kuma, K.-i., and Miyata, T. (2002). Mafft: a novel method for rapid multiple sequence alignment based on fast fourier transform. *Nucleic acids research*, **30**(14), 3059–3066.

Kavya, V. N. S., Tayal, K., Srinivasan, R., and Sivadasan, N. (2019). Sequence alignment on directed graphs. *Journal of Computational Biology*, **26**(1), 53–67.
